# ABO-incompatibility and hepatocellular carcinoma recurrence after living donor liver transplantation: stratified by tumor burden

**DOI:** 10.3389/ti.2026.17121

**Published:** 2026-08-03

**Authors:** Woo-Hyoung Kang, Shin Hwang, Deok-Bog Moon, Ki-Hun Kim, Chul-Soo Ahn, Tae-Yong Ha, Gi-Won Song, Dong-Hwan Jung, Gil-Chun Park, Young-In Yoon, Byeong-Gon Na, Sang-Hoon Kim, Sung-Min Kim, Sung-Gyu Lee

**Affiliations:** Division of Liver Transplantation and Hepatobiliary Surgery, Department of Surgery, Asan Medical Center, University of Ulsan College of Medicine, Seoul, Republic of Korea

**Keywords:** ABO incompatibility, hepatocellular carcinoma (HCC), living donor liver transplantation (LDLT), recurrence, surveillance

## Abstract

ABO-incompatible (ABOi) living donor liver transplantation (LDLT) requires rituximab-based desensitization and intensified early immunosuppression, and whether this promotes hepatocellular carcinoma (HCC) recurrence remains controversial. We hypothesized that this effect depends on tumor burden, assessed morphologically (Milan criteria) and biologically (ADV score). In this single-center study, we analyzed 1,573 adults transplanted for HCC (ABOi, n = 316; ABO-compatible [ABOc], n = 1,257); the primary endpoint was recurrence-free survival (RFS). Crude recurrence was similar (21.8% vs. 20.7%; P = 0.64) despite a lower tumor burden in ABOi recipients. In a propensity score-matched cohort, RFS did not differ overall (hazard ratio 1.11, 95% CI 0.82–1.48), but beyond Milan recurrence was higher in ABOi recipients (53.7% vs. 38.5%; adjusted HR 1.45, 95% CI 1.00–2.09), whereas within Milan it was identical (13.3% vs. 13.4%). The same pattern held for tumor biology: recurrence was higher in ABOi only at ADV ≥5log (64.0% vs. 44.6%; P = 0.022). Higher early tacrolimus exposure in ABOi recipients was associated with recurrence. Overall survival was similar (P = 0.91). ABO-incompatibility did not compromise survival. Recurrence was higher in ABOi recipients with a high tumor burden by either measure, although the formal interaction tests were not significant (P = 0.23 and P = 0.088). These exploratory subgroup findings are hypothesis-generating and support closer early surveillance.

## Graphical Abstract

**Figure:**
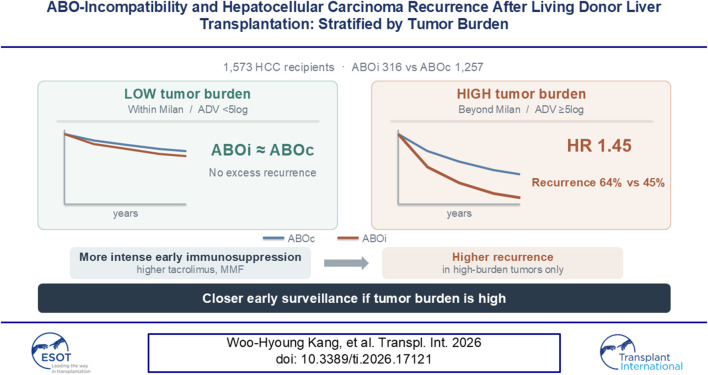

## Introduction

Hepatocellular carcinoma (HCC) is a leading cause of cancer death and the most common oncologic indication for liver transplantation. Transplantation uniquely removes both the tumor and the underlying cirrhotic liver that drives further carcinogenesis [1]. Candidate selection has traditionally been based on the Milan criteria, which identify patients achieving post-transplant survival comparable to that for non-malignant disease [2]. Expanded morphologic criteria and biomarker-based models, such as the up-to-seven criteria and the alpha-fetoprotein (AFP) model, can extend this benefit to selected patients beyond the Milan criteria [3, 4]. In all frameworks, however, tumor burden and biological aggressiveness remain the principal determinants of recurrence, which still occurs in 15%–20% of recipients.

In many Asian countries, including Korea, the shortage of deceased donors means that living donor liver transplantation (LDLT) accounts for most transplant activity [5]. ABOi LDLT expands the living-donor pool for patients who lack an ABO-compatible donor and cannot wait for a deceased-donor graft. Early ABOi transplantation was complicated by frequent antibody-mediated rejection (AMR) and diffuse intrahepatic biliary complications, but rituximab-based desensitization improved outcomes, and contemporary series report survival comparable to ABO-compatible (ABOc) LDLT [6–9]. To overcome the immunologic barrier, ABOi recipients undergo B-cell depletion with rituximab and pretransplant plasma exchange to lower isoagglutinin titers, and they receive more intensive immunosuppression than ABOc recipients during the early postoperative period, when the risk of AMR is highest [6, 8].

This intensified immunosuppression, although necessary for graft survival, raises a biologically plausible oncologic concern. Calcineurin inhibitors can promote tumor invasion and progression through cell-autonomous, transforming growth factor (TGF)-beta-dependent mechanisms independent of host immunity, and the depth of immunosuppression is a recognized, modifiable determinant of post-transplant malignancy and HCC recurrence [10–13]. Conversely, reduced early calcineurin-inhibitor exposure and mammalian target of rapamycin (mTOR) inhibition have been associated with lower recurrence in selected populations [12, 14]. The greater early immunosuppressive burden of ABOi transplantation could therefore impair tumor immunosurveillance when residual tumor cells are most likely to proliferate.

Nevertheless, comparative studies and a recent systematic review with meta-analysis have consistently reported no significant difference in recurrence-free or overall survival between ABOi and ABOc LDLT, supporting ABOi LDLT as an oncologically acceptable option [15, 16]. These analyses, however, treated the transplanted HCC population as a single group and were underpowered within high-risk subgroups, even though the impact of additional immunosuppression should differ between small and large or vascular-invasive tumors. Importantly, data from within ABOi cohorts suggest that the magnitude of the ABOi-specific immunologic burden is oncologically relevant: higher serum tacrolimus levels in the first weeks after transplantation and a greater number of pretransplant therapeutic plasma exchange sessions have each been independently associated with HCC recurrence [17, 18]. Taken together, these findings suggest that any adverse oncologic effect of ABO-incompatibility may be concentrated in patients with biologically aggressive tumors rather than distributed uniformly across the HCC population.

We therefore hypothesized that the effect of ABO-incompatibility depends on tumor burden, with an adverse effect on recurrence emerging in patients with advanced tumors. Because tumor burden comprises both anatomic extent and biological aggressiveness, and the Milan criteria capture only tumor size and number, we used two complementary measures: the morphologic Milan criteria and the biomarker-based ADV score, a composite of AFP, PIVKA-II, and tumor volume. Using a large single-center cohort and propensity score matching, we evaluated the overall effect of ABO-incompatibility on recurrence-free and overall survival, performed stratified analyses by tumor burden assessed both morphologically and biologically, and characterized the timing of recurrence to inform surveillance.

## Materials and methods

### Study population

We retrospectively reviewed consecutive adult recipients (aged ≥18 years) who underwent LDLT for HCC at a single high-volume center between December 2005 and December 2022. Eligibility required viable HCC confirmed on the final explant specimen. Patients with a complete pathologic response, pediatric recipients, recipients of deceased-donor whole-liver grafts, patients undergoing 2:1 (dual-donor) LDLT, and those with incomplete follow-up data were excluded. After applying these criteria, the final cohort comprised 1,573 recipients, of whom 316 (20.1%) underwent ABOi and 1,257 (79.9%) ABOc transplantation. Clinical, operative, and pathologic variables were obtained from a prospectively maintained database and medical records. The study was approved by the Institutional Review Board of [Institution blinded for review], which waived the requirement for informed consent because of the retrospective design and the use of de-identified data, and was conducted in accordance with the Declaration of Helsinki and the Declaration of Istanbul.

### Desensitization and immunosuppression

ABOi LDLT was performed using an institutional rituximab-based desensitization protocol described previously [6]. In brief, a single dose of rituximab (375 mg/m^2^) was given 2–3 weeks before transplantation, and pretransplant plasma exchange was performed as needed to reduce isoagglutinin titers to the institutional target. Local graft infusion and splenectomy, used in earlier eras, were not part of the current protocol. Maintenance immunosuppression in both groups was based on tacrolimus, mycophenolate mofetil (MMF), and a corticosteroid taper, with basiliximab induction used selectively. However, ABOi recipients received more intensive early immunosuppression than ABOc recipients: in addition to higher early tacrolimus trough targets used to reduce the risk of AMR, MMF was added more frequently as part of the institutional ABOi protocol. Serial quantitative measures of immunosuppression exposure were not uniformly available across the study period.

### Definitions, endpoints, and follow-up

Tumor burden was classified by the Milan criteria on explant pathology, and microvascular invasion (MVI) was assessed on the explant by dedicated hepatobiliary pathologists. Serum AFP and protein induced by vitamin K absence-II (PIVKA-II) were recorded at transplantation. Pre-transplant HCC treatment was defined as any bridging or downstaging locoregional therapy before transplantation. To capture tumor biology, tumor burden was additionally quantified using the ADV score, a pre-specified biomarker-based measure validated across hepatic resection and liver transplantation [19–23]. The ADV score is the product of serum AFP (ng/mL), des-gamma-carboxyprothrombin (PIVKA-II, mAU/mL), and tumor volume (mL), expressed on a base-10 logarithmic scale, capturing both biological activity and anatomic extent in a single continuous variable that complements the dichotomous Milan criteria. We applied the previously reported post-transplant cutoff of 5log (corresponding to an ADV product of 100,000), above which the risk of HCC recurrence is substantially increased [19, 20]. After transplantation, surveillance was protocol-driven and identical for ABOi and ABOc recipients. AFP and PIVKA-II were measured at every outpatient visit (monthly during the first year, then every 3 months). Chest and abdomen-pelvis computed tomography were obtained in all recipients at 3 months; thereafter, recipients within the Milan criteria were imaged every 6 months to 3 years, annually to 5 years, and every 2 years thereafter, and those beyond the Milan criteria every 3 months during the first year, every 4–6 months to 3 years, annually to 5 years, and every 2 years thereafter. Imaging frequency was therefore determined by tumor burden, not ABO compatibility. Recurrence was diagnosed primarily by cross-sectional imaging; histologic confirmation was obtained only when clinically indicated and was not routinely required. The primary endpoint, recurrence-free survival (RFS), was the interval from transplantation to first documented recurrence or last follow-up, with patients dying without recurrence censored at death. Overall survival (OS) was defined as the interval from transplantation to death from any cause.

### Statistical analysis

Continuous variables are presented as medians with interquartile ranges (IQR) and were compared using the Mann-Whitney U test; categorical variables are presented as counts with percentages and were compared using the chi-square or Fisher exact test. Recurrence-free and overall survival were estimated using the Kaplan-Meier method and compared using the log-rank test. Hazard ratios (HR) with 95% confidence intervals (CI) were estimated using Cox models; the multivariable RFS model included ABO-incompatibility, recipient age and sex, MELD score, pre-transplant treatment, Milan status, tumor number and largest size, MVI, and log10-transformed AFP and PIVKA-II, with proportional hazards assessed by scaled Schoenfeld residuals. To address the baseline imbalance in tumor burden, 1:2 nearest-neighbor propensity score matching without replacement was performed with a caliper of 0.2 SD of the logit of the propensity score, estimated by logistic regression on tumor and clinical covariates; balance was assessed by standardized mean differences (SMD), with values below 0.10 acceptable. Pre-transplant treatment was included as a covariate rather than as a matching variable to preserve power in the small beyond-Milan subgroup. Stratified analyses by tumor burden were pre-specified. The Milan-based analysis was performed in both the whole and propensity-matched cohorts, whereas the continuous ADV score was analyzed in the whole cohort, where the larger number of high-burden events preserved statistical power. A multiplicative ABO-by-tumor-burden interaction term was fitted in the whole cohort for each measure. Given their exploratory nature, no correction for multiple comparisons was applied. An exploratory mediation analysis used the 1-month tacrolimus concentration as the mediator, with bootstrap confidence intervals. As sensitivity analyses, recurrence was additionally analyzed in a competing-risk framework in which death without recurrence was treated as a competing event, with cumulative incidence functions estimated using the Aalen-Johansen method and subdistribution hazard ratios using Fine-Gray models; alternative ADV cut-offs (3 to 6log) and stratification by transplant era were also examined. All tests were two-sided, and P < 0.05 was considered statistically significant. Analyses were performed using Python (version 3.11) with the lifelines and scikit-learn libraries.

## Results

### Patient characteristics

Of the 1,573 adults with viable HCC on explant pathology, 316 (20.1%) underwent ABOi and 1,257 (79.9%) ABOc transplantation, and the median follow-up was 104 months. The groups were similar in recipient age, sex, underlying liver disease (predominantly hepatitis B virus), graft type, GRWR, AFP, PIVKA-II, and MVI (Table 1). However, ABOi recipients consistently had a lower baseline tumor burden, with a lower MELD score (median 9 vs. 10; P < 0.001), fewer tumors (median 1 vs. 2; P = 0.010), a smaller largest tumor (2.1 vs. 2.3 cm; P = 0.028), a smaller total tumor volume (7.9 vs. 11.2 cm^3^; P = 0.005), and a lower proportion of tumors beyond the Milan criteria (21.2% vs. 29.1%; P = 0.006); they were also more likely to have received pre-transplant locoregional therapy (83.5% vs. 77.3%; P = 0.020). This more favorable tumor profile in the ABOi group, probably reflecting more selective use of ABOi transplantation motivated the matched and stratified analyses.

**TABLE 1:** Baseline characteristics according to ABO compatibility (whole cohort).

Variable	ABO-compatible (n = 1,257)	ABO-incompatible (n = 316)	P value
Recipient age, yr, median (IQR)	55.0 (51.0–60.0)	55.0 (51.0–60.0)	0.735
Male sex, n (%)	1,069 (85.0)	262 (82.9)	0.394
MELD score, median (IQR)	10.0 (8.0–14.0)	9.0 (8.0–12.0)	<0.001
Pre-transplant HCC treatment, n (%)	972 (77.3)	264 (83.5)	0.020
Underlying liver disease, n (%)			
HBV	1,000 (79.6)	249 (78.8)	0.826
HCV	85 (6.8)	27 (8.5)	0.328
Alcoholic	107 (8.5)	24 (7.6)	0.679
Others	65 (5.2)	16 (5.1)	1.000
Graft type, n (%)			
Right lobe	1,161 (92.4)	288 (91.1)	0.545
Left lobe	13 (1.0)	3 (0.9)	1.000
Dual graft	83 (6.6)	25 (7.9)	0.485
GRWR, median (IQR)	1.0 (0.9–1.2)	1.0 (0.9–1.2)	0.423
AFP, ng/mL, median (IQR)	10.5 (4.1–48.0)	9.0 (4.0–34.1)	0.299
PIVKA-II, mAU/mL, median (IQR)	30.0 (19.0–79.0)	31.5 (21.0–66.0)	0.384
Tumor number, median (IQR)	2.0 (1.0–3.0)	1.0 (1.0–2.0)	0.010
Largest tumor size, cm, median (IQR)	2.3 (1.5–3.4)	2.1 (1.5–3.0)	0.028
Total tumor volume, cm^3^, median (IQR)	11.2 (3.1–34.5)	7.9 (1.8–26.8)	0.005
Beyond Milan criteria, n (%)	366 (29.1)	67 (21.2)	0.006
Microvascular invasion, n (%)	220 (17.5)	59 (18.7)	0.686

Data are median (interquartile range) or n (%). MELD, Model for End-Stage Liver Disease; GRWR, graft-to-recipient weight ratio; AFP, alpha-fetoprotein; PIVKA-II, protein induced by vitamin K absence-II. Tumor number, size, volume, and Milan status were assessed on explant pathology. P values from the Mann-Whitney U test (continuous) or chi-square test (categorical).

### Recurrence in the whole and propensity-matched cohorts

**FIGURE 1:**
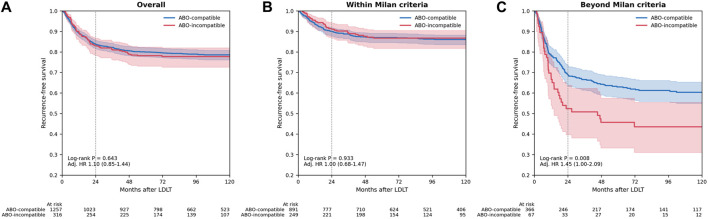
Recurrence-free survival according to ABO compatibility in the whole cohort (n = 1,573). **(A)** Overall, **(B)** within the Milan criteria, and **(C)** beyond the Milan criteria. A significant difference favoring ABO-compatible recipients is seen only beyond the Milan criteria. Shaded areas are 95% confidence intervals; the dashed line marks 24 months.

HCC recurred in 329 of the 1,573 patients (20.9%) during follow-up, comprising 260 ABOc and 69 ABOi recipients. Crude recurrence was similar between the groups (21.8% vs. 20.7%), with 1-, 3-, and 5-year RFS of 89.8%/81.3%/78.2% versus 89.1%/82.2%/80.0%, respectively (log-rank P = 0.64; Figure 1A). In univariable analysis, ABO-incompatibility was not associated with recurrence (HR 1.06, 95% CI 0.82–1.39; Table 2). After adjustment for clinical and tumor factors, the point estimate increased toward higher risk (HR 1.19, 95% CI 0.91–1.55; P = 0.21), a shift that becomes apparent only after the more favorable baseline tumor burden of the ABOi group is taken into account. Beyond-Milan status, MVI, pre-transplant treatment, tumor number and size, AFP, and PIVKA-II were the dominant independent predictors of recurrence (Table 2).

**TABLE 2:** Univariable and multivariable Cox regression for recurrence-free survival (whole cohort).

Variable	Univariable HR (95% CI), P	Multivariable HR (95% CI), P
ABO-incompatibility	1.06 (0.82–1.39), 0.648	1.19 (0.91–1.55), 0.210
Recipient age, per yr	0.98 (0.96–0.99), <0.001	1.00 (0.98–1.01), 0.818
Male sex	1.73 (1.21–2.49), 0.003	1.52 (1.05–2.19), 0.025
MELD, per point	0.99 (0.97–1.01), 0.304	0.98 (0.96–1.00), 0.099
Pre-transplant treatment	2.41 (1.71–3.39), <0.001	1.68 (1.18–2.40), 0.004
Beyond Milan criteria	3.69 (2.97–4.58), <0.001	1.70 (1.28–2.25), <0.001
Tumor number, per lesion	1.11 (1.09–1.13), <0.001	1.04 (1.01–1.07), 0.003
Largest tumor size, per cm	1.24 (1.21–1.28), <0.001	1.10 (1.05–1.16), <0.001
Microvascular invasion	5.53 (4.45–6.87), <0.001	3.11 (2.41–4.00), <0.001
log10 AFP	1.76 (1.57–1.97), <0.001	1.25 (1.10–1.40), <0.001
log10 PIVKA-II	1.91 (1.67–2.18), <0.001	1.17 (1.00–1.38), 0.053

HR, hazard ratio; CI, confidence interval. The multivariable model included all listed variables (n=1,565; 329 events). AFP and PIVKA-II were modeled as log10-transformed continuous variables.

**FIGURE 2:**
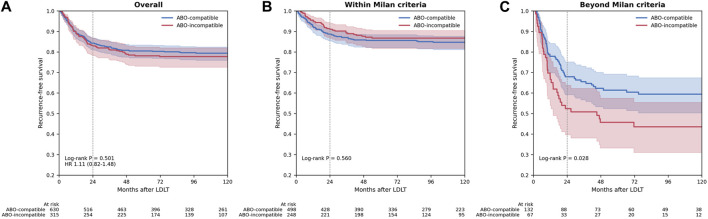
Recurrence-free survival according to ABO compatibility in the 1:2 propensity score-matched cohort (ABOi 315: ABOc 630). **(A)** Overall, **(B)** within the Milan criteria, and **(C)** beyond the Milan criteria. After matching, the difference beyond the Milan criteria persists, whereas overall and within-Milan outcomes remain comparable. Shaded areas are 95% confidence intervals; the dashed line marks 24 months.

To address the baseline imbalance in tumor burden, a 1:2 propensity-matched cohort was constructed (ABOi 315: ABOc 630), with excellent covariate balance (all SMD <0.05; Table 3). In the matched cohort, RFS again did not differ between groups (5-year RFS 78.1% vs. 80.5%; log-rank P = 0.50; HR 1.11, 95% CI 0.82–1.48), confirming the absence of an overall adverse effect of ABO-incompatibility (Figure 2A).

**TABLE 3:** Propensity score-matched cohort and covariate balance before and after matching.

Covariate	ABO-compatible (n = 630)	ABO-incompatible (n = 315)	SMD before	SMD after
Beyond Milan criteria, %	21.0	21.3	−0.183	+0.008
Microvascular invasion, %	17.3	18.7	+0.030	+0.037
log10 AFP	1.00	1.00	−0.089	+0.014
log10 PIVKA-II	1.50	1.50	−0.021	+0.045
Recipient age, yr	55.0	55.0	−0.037	−0.013
Male sex, %	84.3	82.9	−0.058	−0.039
MELD score	9.0	9.0	−0.267	+0.032

Values are matched-group medians or percentages. SMD, standardized mean difference; a |SMD| <0.10 indicates acceptable balance. Covariates were matched 1:2 by nearest-neighbor matching on the logit of the propensity score (caliper 0.2 SD).

### Overall survival

Overall survival was also similar between the groups. A total of 410 deaths occurred during follow-up (333 ABOc and 77 ABOi), and the 1-, 3-, and 5-year overall survival rates were 95.6%/85.7%/81.2% in the ABOc group and 95.3%/86.4%/81.4% in the ABOi group (log-rank P = 0.91). Thus, in the whole cohort, ABO-incompatibility compromised neither recurrence-free nor overall survival, consistent with contemporary ABOi LDLT being oncologically safe on average.

### Tumor morphology (Milan criteria)

The effect of ABO-incompatibility differed according to tumor stage (Table 4). Among the 1,140 patients within the Milan criteria, 152 recurrences occurred (119 ABOc and 33 ABOi), and recurrence was nearly identical between groups (ABOi 13.3% vs. ABOc 13.4%; 5-year RFS 86.7% vs. 87.0%; log-rank P = 0.93; adjusted HR 1.00, 95% CI 0.68–1.47; Figure 1B), and this equivalence was maintained in the propensity-matched cohort (Figure 2B). Among the 433 patients beyond the Milan criteria, 177 recurrences occurred (141 ABOc and 36 ABOi), and recurrence was significantly higher in ABOi recipients (53.7% vs. 38.5%; 5-year RFS 45.7% vs. 63.0%; log-rank P = 0.008; adjusted HR 1.45, 95% CI 1.00–2.09; P = 0.049; Figure 1C). This difference persisted in the propensity-matched cohort (ABOi 53.7% vs. ABOc 40.2%; log-rank P = 0.028; Figure 2C). The multiplicative ABO-by-Milan interaction term was directionally consistent but not significant (HR 1.39; P = 0.23), reflecting the limited events in this subgroup (36 recurrences in 67 patients); the stratum-specific estimates are therefore best interpreted as a consistent gradient.

**TABLE 4:** Recurrence and recurrence-free survival by ABO compatibility, stratified by the Milan criteria.

Stratum	ABOc: recurrence % (5-year RFS %)	ABOi: recurrence % (5-year RFS %)	Log-rank P	Adjusted HR for ABOi (95% CI), P
Overall (n = 1,573)	20.7 (80.0)	21.8 (78.2)	0.643	1.10 (0.85–1.44), 0.466
Within Milan (n = 1,140)	13.4 (87.0)	13.3 (86.7)	0.933	1.00 (0.68–1.47), 0.982
Beyond Milan (n = 433)	38.5 (63.0)	53.7 (45.7)	0.008	1.45 (1.00–2.09), 0.049

RFS, recurrence-free survival; HR, hazard ratio; ABOc, ABO-compatible; ABOi, ABO-incompatible. Adjusted HR for ABO-incompatibility within each stratum was derived from a Cox model including microvascular invasion, pre-transplant treatment, and log10-transformed AFP and PIVKA-II. The multiplicative ABOi-by-Milan interaction term was not statistically significant (P=0.23).

### Tumor biology (ADV score)

**FIGURE 3:**
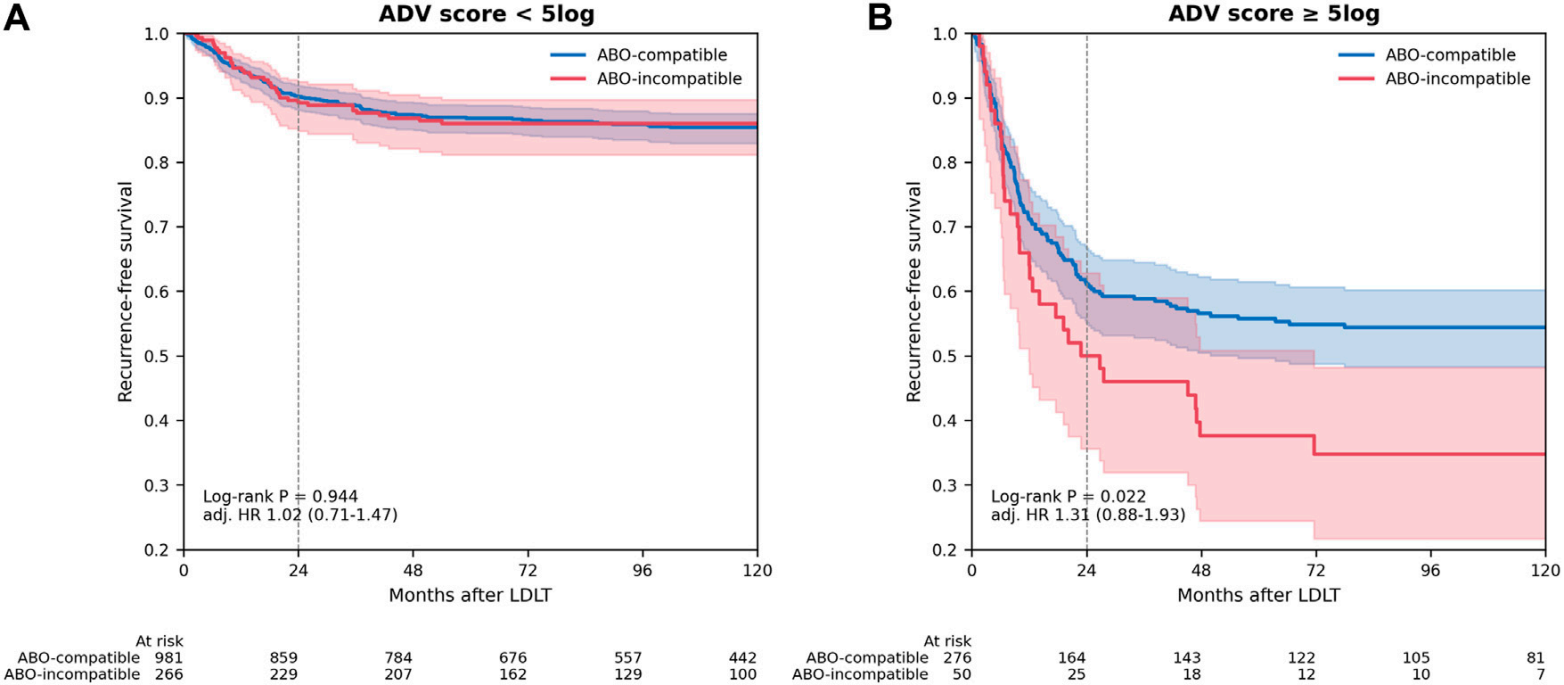
Recurrence-free survival according to ABO compatibility, stratified by the ADV score in the whole cohort. **(A)** ADV score below 5log and **(B)** ADV score of 5log or higher. A difference favoring ABO-compatible recipients is seen only in the high-ADV stratum. Shaded areas are 95% confidence intervals; the dashed line marks 24 months.

Because the Milan criteria reflect only tumor size and number, we next performed a stratified analysis using a complementary, pre-specified measure of tumor biology, the AFP-DCP-volume (ADV) score, which combines serum tumor markers with tumor volume, applying the established post-transplant cutoff of 5log [19, 20]. The results were directionally consistent with the Milan-based analysis (Table 5; Figure 3). Among patients with an ADV score below 5log, recurrence was nearly identical between groups (ABOc 14.0% vs. ABOi 13.9%; log-rank P = 0.94). Among patients with an ADV score of 5log or higher, recurrence was higher in ABOi recipients (64.0% vs. 44.6%; 5-year RFS 37.6% vs. 55.7%; log-rank P = 0.022), although this difference attenuated after adjustment for microvascular invasion and pre-transplant treatment (HR 1.31, 95% CI 0.88–1.93; P = 0.18). The continuous ADV-by-ABO interaction term approached significance (P = 0.088), and overall survival did not differ within either ADV stratum (ADV≥5log, log-rank P = 0.29). Thus, the tumor-burden-dependent effect of ABO-incompatibility was observed with an independent, biomarker-based measure of tumor burden as well as with the Milan criteria, although it remained statistically borderline. In a competing-risk analysis treating death without recurrence as a competing event, the findings were unchanged (beyond Milan: subdistribution HR 1.54, 95% CI 1.08–2.19; ADV ≥5log: 1.58, 95% CI 1.07–2.32; Supplementary Table S1). The direction of effect was consistent across ADV cut-offs of 3 to 6log, reaching significance only at the pre-specified 5log threshold (Supplementary Table S2), and the results did not differ by transplant era (ABO-by-era interaction P = 0.52; Supplementary Table S3).

**TABLE 5:** Recurrence and recurrence-free survival by ABO compatibility, stratified by the ADV score (whole cohort).

Stratum	ABOc: recurrence % (5-year RFS %)	ABOi: recurrence % (5-year RFS %)	Log-rank P	Adjusted HR for ABOi (95% CI), P
ADV score <5log (n = 1,247)	14.0 (86.8)	13.9 (86.0)	0.944	1.02 (0.71–1.47), 0.921
ADV score ≥5log (n = 326)	44.6 (55.7)	64.0 (37.6)	0.022	1.31 (0.88–1.93), 0.180

RFS, recurrence-free survival; HR, hazard ratio; ADV, alpha-fetoprotein-des-gamma-carboxyprothrombin-tumor volume score. The 5log cutoff is the previously reported post-transplant threshold [19, 20]. Adjusted HR was derived from a Cox model including microvascular invasion and pre-transplant treatment; AFP, PIVKA-II, and tumor volume were not added as separate covariates because they constitute the ADV score. The continuous ADV-by-ABO interaction term was not statistically significant (P=0.088).

### Timing of recurrence

Recurrences occurred early in both groups. Among patients who recurred, the median time to recurrence was similar (ABOi 12.6 vs. ABOc 11.7 months; P = 0.37), and approximately 77% of all recurrences occurred within 24 months in both groups (76.8% vs. 77.3%). ABO-incompatibility was therefore not associated with earlier recurrence. The absolute early-recurrence burden was highest in beyond-Milan ABOi recipients (24-month recurrence 46.3% vs. 30.9%), identifying the main target for intensified surveillance.

### Early tacrolimus exposure and recurrence

To explore the proposed mechanism, we examined tacrolimus trough concentrations at 1 and 3 months after transplantation, available in 1,532 and 1,526 patients, respectively. Consistent with the more intensive ABOi protocol, ABOi recipients had higher levels than ABOc recipients at both 1 month (9.5 vs. 9.1 ng/mL; P < 0.001) and 3 months (8.9 vs. 8.5; P < 0.001). A higher 1-month tacrolimus concentration was independently associated with recurrence in the whole cohort (adjusted HR 1.09 per ng/mL, 95% CI 1.01–1.19; P = 0.03). This association was largely confined to high tumor burden, being apparent beyond the Milan criteria (HR 1.12; P = 0.06) and at ADV ≥ 5log (HR 1.11; P = 0.09) but not within Milan (HR 1.05; P = 0.39), although the tacrolimus-by-Milan interaction was not significant (P = 0.54). In an exploratory mediation analysis, the 1-month tacrolimus concentration accounted for only a small, statistically imprecise fraction of the ABO-incompatibility effect, modestly attenuating its estimate when added to the model. The association was weaker in the smaller propensity-matched cohort (HR 1.09; P = 0.12).

## Discussion

In this large single-center cohort of HCC LDLT recipients, ABO-incompatibility had no detectable effect on recurrence in the overall population, a finding that was robust to multivariable adjustment and propensity score matching and that is consistent with previous comparative studies and a recent meta-analysis [15, 16]. The main observation of this study is that this overall null result may conceal clinically relevant heterogeneity, with the effect of ABO-incompatibility appearing to depend on tumor burden, whether assessed by tumor morphology (Milan criteria) or by tumor biology (the marker-based ADV score). Within the Milan criteria, recurrence was nearly identical between ABOi and ABOc recipients, whereas beyond the Milan criteria, ABO-incompatibility was associated with a significantly higher recurrence risk that persisted after propensity matching. To our knowledge, this is the first study to suggest that the oncologic risk of ABO-incompatibility may depend on tumor burden. Given the borderline beyond-Milan estimate and the non-significant interaction, however, this observation is hypothesis-generating.

This tumor-burden dependence is biologically plausible. ABOi recipients receive a greater early immunosuppressive load than ABOc recipients—B-cell depletion with rituximab, pretransplant plasma exchange, higher early calcineurin-inhibitor targets, and more frequent mycophenolate mofetil—all required to prevent antibody-mediated rejection [6, 8]. Because the cumulative depth of immunosuppression is a recognized, modifiable determinant of post-transplant HCC recurrence [10, 11, 13, 14], this additional burden would be expected to matter only when residual tumor is present: inconsequential in low-burden tumors, consistent with the identical within-Milan outcomes observed here, but of potential relevance beyond the Milan criteria, where micrometastatic disease is more likely and the balance between residual tumor and immune control is precarious.

Our own mechanistic evidence for this pathway is, however, limited. The between-group difference in early tacrolimus exposure was small (1-month trough 9.5 vs. 9.1 ng/mL), and although a higher 1-month level was independently associated with recurrence (adjusted HR 1.09 per ng/mL), particularly at high tumor burden, it accounted for only a small and imprecise fraction of the ABO-incompatibility effect in mediation analysis. Because the greater immunosuppression in ABOi recipients reflects not only tacrolimus but also more frequent mycophenolate mofetil use, this single-agent measure probably underestimates the total immunosuppressive load. The effect may therefore relate to the net intensity of immunosuppression rather than the humoral alloimmune challenge itself—B-cell depletion with rituximab has not been shown to increase HCC recurrence [24], and the baseline isoagglutinin titer reflects the antibody barrier rather than a direct pro-tumoral stimulus—and is supported by independent ABOi cohorts in which recurrence tracked with early tacrolimus exposure and plasma-exchange burden [17, 18] and by an early series in which recurrence was concentrated beyond the Milan criteria [25]. These observations are consistent with, but do not establish, a causal pathway. Clinically, the principal modifiable factor in high-risk ABOi recipients may be the depth of maintenance immunosuppression rather than the desensitization itself, and this lever is most applicable beyond the early postoperative period. The proposed mechanism is hypothesis-generating and was not formally tested in this study.

The timing of recurrence has direct implications for surveillance. Recurrence occurred predominantly within the first two postoperative years in both groups, and ABO-incompatibility was not associated with earlier recurrence; the signal is one of absolute risk rather than accelerated timing, with beyond-Milan ABOi recipients carrying the highest early-recurrence burden and nearly half recurring within 24 months. These patients are strong candidates for intensified early surveillance. Under our current protocol, beyond-Milan recipients undergo imaging every 3 months during the first year and every 4–6 months thereafter; in high-burden ABOi recipients we suggest extending the 3-monthly interval through the second post-transplant year, with tumor markers at each visit, before returning to the standard schedule. This is a modest intensification of an existing protocol and should be regarded as a testable proposal. In ABOi recipients, higher early calcineurin-inhibitor exposure is largely unavoidable, constraining any early reduction in immunosuppression [12]. mTOR inhibitors may also attenuate the endothelial injury underlying antibody-mediated rejection, offering a potential dual benefit in ABOi recipients: mitigating alloimmune endothelial damage while permitting reduced calcineurin-inhibitor exposure. They have historically been avoided in the first postoperative months because of concerns about wound healing and hepatic artery thrombosis, but this concern is less clearly supported by contemporary data. Our practice has been to transition after the third month; the present findings suggest this timing warrants reconsideration in high-burden ABOi recipients, although the SiLVER trial showed benefit mainly in lower-risk patients [14]. Post-transplant risk scores such as RETREAT may help refine surveillance [26, 27]. Although overall survival did not differ, earlier detection remains clinically meaningful, enabling curative-intent or timely systemic treatment.

A further consideration is that tumor burden was defined on explant pathology, which provides the most accurate assessment of tumor extent but limits direct translation to preoperative candidate selection. Notably, the ADV score is partly preoperative by construction: alpha-fetoprotein and PIVKA-II are measured before transplantation, and both retain long-term prognostic value after living donor liver transplantation [23], and tumor volume can be estimated from pre-transplant cross-sectional imaging, so a pre-transplant version is computable in principle. Systematic pre-transplant volumetry was unavailable in our database; prospective validation of an imaging-based measure is therefore the logical next step, and until then our findings are best applied to post-transplant surveillance and immunosuppression rather than to candidate selection.

Our findings extend, rather than contradict, the existing literature. Previous series and a recent meta-analysis found no adverse oncologic effect of ABO-incompatibility, but did not formally test whether this effect varies with tumor burden or use a biologic measure of aggressiveness, and were underpowered for high-risk subgroups [15, 16]; our overall results are fully consistent with theirs. Independent series, including one combining rituximab with total plasma exchange, likewise identified AFP, tumor size, encapsulation, and microvascular invasion, not ABO status, as the relevant risk factors [24]. Using a large cohort with propensity matching and pre-specified stratified analyses, however, we found that the average safety of ABOi LDLT may conceal a clinically meaningful gradient. The consistency of this gradient across two conceptually distinct measures of tumor burden, the morphology-based Milan criteria and the marker-based ADV score, argues against a chance subgroup effect, although both analyses remained borderline [19, 20].

Strengths include the large size, explant-based pathologic staging, long follow-up, and a propensity-matched design. Limitations should also be acknowledged. First, the retrospective, single-center design carries risks of selection and residual confounding, although matching achieved excellent covariate balance. Second, the overall immunosuppressive load, including mycophenolate mofetil dosing and cumulative exposure, was not quantified, so the mechanistic and mediation analyses are exploratory. Third, the interaction term was not significant, reflecting limited events in the beyond-Milan ABOi subgroup, so the subgroup findings are hypothesis-generating and warrant external validation. Fourth, tumor burden was classified on explant pathology. Prospective studies with detailed immunosuppression-exposure data are needed to confirm the mechanism and define optimal strategies for this subgroup.

In conclusion, ABO-incompatibility did not adversely affect HCC recurrence overall but differed according to tumor burden, with a higher recurrence risk in patients with advanced tumors, defined by either the Milan criteria or the ADV score. Overall survival did not differ in any stratum. Because the formal interaction tests were not significant, these subgroup findings are hypothesis-generating and require external validation. Nevertheless, given that recurrences occur predominantly within the first two post-transplant years, ABOi recipients with advanced tumors may warrant intensified early surveillance, together with consideration of a calcineurin-inhibitor-sparing, mTOR inhibitor-based regimen beyond the early postoperative period.

## Data Availability

The raw data supporting the conclusions of this article will be made available by the authors, without undue reservation.
