# Supplementary material for: ABO-incompatibility and hepatocellular carcinoma recurrence after living donor liver transplantation: stratified by tumor burden
Source: Transpl Int. 2026 Aug 3;39:17121. doi: 10.3389/ti.2026.17121 (PMC13478018; doi:10.3389/ti.2026.17121)
Supplement: Supplementary file 1 [file Table1.docx]

**Supplementary Table S1. Competing-risk analysis of hepatocellular carcinoma recurrence according to ABO compatibility**

| **Subgroup** | **n (ABOc / ABOi)** | **2-year CIF, %** | **5-year CIF, %** | **10-year CIF, %** | **sHR (95% CI)** | **P value** |
| --- | --- | --- | --- | --- | --- | --- |
| Overall | 1257 / 316 | 15.9 / 16.8 | 19.6 / 21.3 | 20.9 / 21.7 | 1.05 (0.83–1.33) | 0.690 |
| Within Milan | 891 / 249 | 9.9 / 8.4 | 12.7 / 13.0 | 13.6 / 13.0 | 0.99 (0.70–1.39) | 0.952 |
| Beyond Milan | 366 / 67 | 30.9 / 46.3 | 36.4 / 52.5 | 38.8 / 54.6 | 1.54 (1.08–2.19) | 0.018 |
| ADV <5log | 981 / 266 | 9.7 / 10.5 | 12.9 / 13.6 | 14.2 / 13.6 | 1.01 (0.74–1.37) | 0.970 |
| ADV ≥5log | 276 / 50 | 38.4 / 50.0 | 43.5 / 62.0 | 44.8 / 64.8 | 1.58 (1.07–2.32) | 0.020 |
| Beyond Milan, adjusted* | — | — | — | — | 1.37 (0.96–1.96) | 0.081 |
| ADV ≥5log, adjusted* | — | — | — | — | 1.33 (0.91–1.97) | 0.144 |

Values for cumulative incidence functions are given as ABO-compatible / ABO-incompatible. Death without recurrence was treated as a competing event (329 recurrences, 135 competing deaths, and 1,109 censored observations). Cumulative incidence functions were estimated using the Aalen–Johansen method, and subdistribution hazard ratios for ABO-incompatibility versus ABO-compatibility were estimated using Fine–Gray models.

*Adjusted for microvascular invasion and pre-transplant treatment.

ABOc, ABO-compatible; ABOi, ABO-incompatible; ADV, alpha-fetoprotein–des-gamma-carboxyprothrombin–tumor volume; CI, confidence interval; CIF, cumulative incidence function; sHR, subdistribution hazard ratio.

**Supplementary Table S2. Sensitivity analysis of the effect of ABO-incompatibility using alternative ADV score cut-offs**

| **ADV cut-off** | **High-ADV stratum, n (events)** | **HR (95% CI)** | **P value** | **Low-ADV stratum, n (events)** | **HR (95% CI)** | **P value** |
| --- | --- | --- | --- | --- | --- | --- |
| 3 log | 1085 (294) | 1.22 (0.93–1.61) | 0.143 | 488 (35) | 0.52 (0.21–1.27) | 0.148 |
| 4 log | 637 (221) | 1.29 (0.93–1.77) | 0.122 | 936 (108) | 0.94 (0.60–1.47) | 0.792 |
| 5 log | 326 (155) | 1.56 (1.06–2.30) | 0.024 | 1247 (174) | 1.01 (0.71–1.44) | 0.946 |
| 6 log | 164 (92) | 1.41 (0.83–2.37) | 0.203 | 1409 (237) | 1.09 (0.81–1.47) | 0.567 |

Hazard ratios are for ABO-incompatibility versus ABO-compatibility within each stratum, estimated using unadjusted Cox proportional hazards models. The direction of effect in the high-ADV stratum was consistent across all cut-offs examined, and no difference was observed in any low-ADV stratum. Statistical significance was reached only at the pre-specified 5 log threshold. At lower cut-offs the high-ADV stratum becomes progressively diluted with lower-risk patients, whereas at the highest cut-off the stratum becomes small.

ADV, alpha-fetoprotein–des-gamma-carboxyprothrombin–tumor volume; CI, confidence interval; HR, hazard ratio.

**Supplementary Table S3. Era-stratified analysis of the effect of ABO-incompatibility on hepatocellular carcinoma recurrence**

| **Analysis** | **2005–2014, n (events)** | **HR (95% CI)** | **P value** | **2015–2022, n (events)** | **HR (95% CI)** | **P value** |
| --- | --- | --- | --- | --- | --- | --- |
| Whole cohort | 749 (180) | 1.21 (0.83–1.75) | 0.321 | 824 (149) | 1.01 (0.70–1.46) | 0.956 |
| Beyond Milan | 228 (105) | 1.99 (1.23–3.24) | 0.005 | 205 (72) | 1.41 (0.81–2.44) | 0.226 |

Hazard ratios are for ABO-incompatibility versus ABO-compatibility, estimated using unadjusted Cox proportional hazards models. The ABO-by-era interaction term was not statistically significant (P = 0.52), indicating that the findings were not attributable to an era effect. The proportion of ABO-incompatible transplants increased from 15.9% in the earlier era to 23.9% in the later era.

CI, confidence interval; HR, hazard ratio.
